# An *arginase 2* promoter transgenic line illuminates immune cell polarisation in zebrafish

**DOI:** 10.1242/dmm.049966

**Published:** 2023-05-03

**Authors:** Ffion R. Hammond, Amy Lewis, Zoë C. Speirs, Holly E. Anderson, Tamara Sipka, Lewis G. Williams, Mai Nguyen-Chi, Annemarie H. Meijer, Geert F. Wiegertjes, Philip M. Elks

**Affiliations:** ^1^ The Bateson Centre, Department of Infection and Immunity and Cardiovascular Disease, University of Sheffield, Western Bank, Sheffield S10 2TN, UK; ^2^ LPHI, University of Montpellier, CNRS, 34090 Montpellier, France; ^3^ Institute of Biology Leiden, Leiden University, Einsteinweg 55, 2333 CC Leiden, The Netherlands; ^4^ Aquaculture and Fisheries Group, Department of Animal Sciences, Wageningen University & Research, 6700 AH Wageningen, The Netherlands

**Keywords:** Anti-inflammatory, Infection, Inflammation, Macrophage, Neutrophil, Zebrafish

## Abstract

Innate immune responses to inflammation and infection are complex and represent major challenges for developing much needed new treatments for chronic inflammatory diseases and drug-resistant infections. To be ultimately successful, the immune response must be balanced to allow pathogen clearance without excess tissue damage, processes controlled by pro- and anti-inflammatory signals. The roles of anti-inflammatory signalling in raising an appropriate immune response are underappreciated, representing overlooked potential drug targets. This is especially true in neutrophils, a difficult cell type to study *ex vivo* owing to a short lifespan, dogmatically seen as being highly pro-inflammatory. Here, we have generated and describe the first zebrafish transgenic line [*TgBAC(arg2:eGFP)sh571*] that labels expression of the anti-inflammatory gene *arginase 2* (*arg2*) and show that a subpopulation of neutrophils upregulate arginase soon after immune challenge with injury and infection. At wound-healing stages, *arg2:GFP* is expressed in subsets of neutrophils and macrophages, potentially representing anti-inflammatory, polarised immune cell populations. Our findings identify nuanced responses to immune challenge *in vivo,* responses that represent new opportunities for therapeutic interventions during inflammation and infection.

## Teaser

**Summary:** A new zebrafish transgenic line that labels the expression of the gene *arg2* can be used to visualise immune cell polarisation and investigate the mechanisms of innate immunity during infections.

## INTRODUCTION

Initial immune responses to inflammatory or infection stimuli are mediated by innate immune cells, of which leukocytes are major players. Two important leukocyte cell types, neutrophils and macrophages, work together to neutralise invading threats while promoting tissue healing and restoration of homeostasis. Neutrophils and macrophages have evolved together and co-exist in invertebrate and vertebrate species (Hartenstein, 2006). Neutrophils are often observed to be the first immune cell type to respond to immune challenge, rapidly migrating towards the stimuli and becoming activated to a pro-inflammatory and antimicrobial state, clearing damaged cells and invading pathogens. Macrophages also rapidly respond to immune challenge, with pro-inflammatory phenotypes emerging soon after immune challenge aiding microbial clearance (often termed M1 or classically activated). Dogmatically, neutrophils are considered a blunt first line of defence that, once activated, remain so until cleared from tissues, either by apoptosis and subsequent efferocytosis by macrophages or by migration (Duffin et al., 2010; Elks et al., 2011b; Mathias et al., 2006). Persistence of neutrophils during disease can cause considerable bystander damage to healthy surrounding tissues driving chronic pathologies. Macrophage phenotypes, on the other hand, are well characterised as being plastic throughout the pathogenesis of inflammation and infections in a process termed macrophage polarisation. Pro-inflammatory macrophage phenotypes are followed by anti-inflammatory phenotypes that promote healing and restoration of homeostasis (often termed M2 or alternatively activated). Many of the above observations come from *in vitro* approaches. *In vivo* exploration indicates that macrophage polarisation is not binary but rather a spectrum of phenotypes and behaviours, and that the neutrophil response is more plastic than previously thought, with emerging roles for neutrophil subsets in tissue protection and healing (Ballesteros et al., 2020; Giese et al., 2019; Phillipson and Kubes, 2019).

Both neutrophils and macrophages are activated by danger- and pathogen-associated molecular patterns (DAMPs and PAMPs, respectively), which trigger the production of pro-inflammatory signals (e.g. IL-1β and TNF-α) after immune challenge (Kato and Kitagawa, 2006; Shapouri-Moghaddam et al., 2018). These activate the production of antimicrobial molecules, including reactive nitrogen species (RNS), via the enzyme inducible nitric oxide synthase (iNOS) (Stuehr and Marletta, 1985). Immunomodulatory signals are required to limit and eventually turn off the pro-inflammatory response for tissues to regain homeostasis. The best characterised of these anti-inflammatory signalling molecules are cytokines, including the interleukins (ILs) IL-4, IL-13 and IL-10, alongside proteins that dampen the pro-inflammatory response, including arginase (Wiegertjes et al., 2016). Despite their classification as anti-inflammatory, these signals have also been identified as being upregulated in some pro-inflammatory situations (Munder et al., 2005). The production of antimicrobial RNS is negatively regulated by the enzyme arginase. Arginase competes with iNOS for a shared substrate, L-arginine, a limited resource within the cell. The immunomodulatory properties of arginase extend beyond the regulation of RNS production. Arginase 2 is essential for IL-10-mediated downregulation of pro-inflammatory factors, making it a key anti-inflammatory enzyme (Dowling et al., 2021). Arginase and iNOS have been well characterised in murine models as being strongly expressed in macrophage subtypes, with arginase being a wound-healing/anti-inflammatory macrophage marker and iNOS a pro-inflammatory macrophage marker (Munder et al., 1998). In human macrophages, the distinction between iNOS and arginase-expressing macrophage subtypes is less well defined, due, in part, to lower macrophage RNS in humans compared to that in mice. Interestingly, human neutrophils constitutively express arginase with levels increasing after infection *in vitro*, but its roles are poorly understood *in vivo* (Munder, 2009). Considering that arginase is not expressed highly by murine neutrophils (El Kasmi et al., 2008; MacMicking et al., 1997), alternative *in vivo* models are required to investigate the roles of arginase in immunomodulation and human disease.

The zebrafish has become a powerful model organism to determine the molecular mediators of immunity against injury and pathogenic challenges (Henry et al., 2013; Yoshida et al., 2017). Advantages of the zebrafish model include transparent larvae combined with fluorescent transgenic lines, allowing detailed microscopy in an intact organism *in vivo*. A key turning point in zebrafish immunity research came with the development of transgenic lines marking neutrophil and macrophage cell populations. Initially, transgenic lines were developed that labelled whole populations of immune cells in zebrafish larvae, e.g. *TgBAC(mpx:GFP)i114* labelling the total neutrophil population and *Tg(mpeg1:GFP)* labelling the total macrophage population (Ellett et al., 2011; Renshaw et al., 2006). More recently, transgenic lines of important pro-inflammatory cytokines have become available [e.g. *TgBAC(tnfa:GFP)pd1028* and *TgBAC(il-1beta)sh445*], allowing in-depth analysis of the cells producing these important signals following a variety of immune challenges *in vivo* (Marjoram et al., 2015; Ogryzko et al., 2019). These transgenic lines utilised bacterial artificial chromosome (BAC) technology, which allows tens of kilobases of promoter region to be used to drive expression of the fluorescent protein, ensuring that the expression of the transgene recapitulates endogenous expression patterns as closely as possible. A key gap in this zebrafish ‘transgenic toolbox’ is an anti-inflammatory fluorescent transgenic line. We therefore set out to develop an *arginase 2* (*arg2*) promoter-driven BAC transgenic line to understand the arginase response to immune challenge *in vivo*.

Here, we compared existing expression data of arginase genes in zebrafish neutrophils and macrophages and show that *arg2* is the most highly expressed arginase in zebrafish immune cells. We developed a new BAC transgenic line that drives GFP under the control of the *arg2* promoter and demonstrate that the *arg2:GFP* transgene is expressed in ionocytes, a population of skin-resident cells, in resting conditions. Following a range of immune challenges, including tailfin transection (sterile injury) and *Mycobacterium marinum* (bacterial) and *Cryptococcus neoformans*/*Candida albicans* (fungal) infections, *arg2:GFP* expression was predominantly upregulated in neutrophils. We identify a small population of macrophages that express *arg2* after injury and infection, suggesting the presence of anti-inflammatory macrophages in zebrafish. The *arg2:GFP* transgenic line has the potential to uncover new mechanisms behind innate immune regulation during *in vivo* immune challenge.

## RESULTS

### *TgBAC(arg2:GFP)sh571* is expressed in ionocytes in resting conditions

There are two isozymes of arginase in most mammals and fish, arginase 1 (ARG1) and arginase 2 (ARG2). In mice, ARG1 and ARG2 are both expressed by macrophages; however, it is ARG1 that is the most widely studied in macrophage polarisation, with increased expression of cytosolic ARG1 protein depleting intracellular stores of arginine (Rath et al., 2014). In fish, arginases have been studied in the common carp (*Cyprinus carpio*), a species phylogenetically close to zebrafish, in which *arg2* is the most highly expressed gene of the arginase family in immune cells (Wentzel et al., 2020a; Wiegertjes et al., 2016). Head-kidney-derived macrophages of carp that have been polarised towards anti-inflammatory phenotypes using cAMP have a 16-fold upregulation of *arg2* expression (Wentzel et al., 2020b; Wiegertjes et al., 2016).

Zebrafish have orthologues of mammalian ARG1 and ARG2 that share strong sequence homology with human (and mouse) orthologues (Fig. S1A-C). Zebrafish *arg1* (NCBI accession number NM_001045197) and *arg2* (NCBI accession number NM_199611) were compared using existing transcriptomics datasets to identify innate immune cell expression. Fluorescence-activated cell sorting (FACS)-purified neutrophils [*Tg(mpx:GFP)i114*] and macrophages [*Tg(mpeg1:Gal4-VP16)gl24/(UAS-E1b:Kaede)s1999t*] from unchallenged 5 days post fertilisation (dpf) larvae both expressed *arg2*, whereas *arginase 1* (*arg1*) was not expressed at detectable levels (Fig. S1D,E; using raw data from Rougeot et al., 2019). *arg2* expression was found at high levels in the bulk, non-immune-cell population and it was also expressed in the immune cell populations (Fig. S1F,G; data from Rougeot et al., 2019). *arg2* expression was approximately 1.5-fold higher in neutrophils than that in macrophages in unchallenged zebrafish larvae (Fig. S1F,G; using raw data from Rougeot et al., 2019). In order to assess relative levels of *arg2* expression in immune cells of zebrafish larvae, cDNA from FACS-isolated macrophages and neutrophils was analysed by real-time quantitative PCR (RT-qPCR) (Fig. S1H-J) (Nguyen-Chi et al., 2014). Sorted neutrophils expressed higher levels of *arg2* compared to *arg2* expression in sorted macrophages and the background of the larvae (Fig. S1J). In unchallenged adult zebrafish head kidneys (the site of haematopoiesis), single-cell RNA sequencing (RNAseq) identified *arg2* expression in a population of neutrophils, a smaller population of monocytes/macrophages, with no detectable expression in other blood lineages (thrombocytes/erythrocytes) (Fig. S1K,L; data from The Zebrafish Blood Atlas, https://www.sanger.ac.uk/science/tools/basicz/basicz/; Athanasiadis et al., 2017). From the same dataset, *arg1* expression was not detected in any immune cell lineage (Fig. S1K,L).

Based on the predominant expression of *arg2* in fish innate immune cells, we chose to develop an *arg2* transgenic zebrafish line to investigate its expression during immune challenge *in vivo*. We adopted a BAC transgenesis approach, using a BAC (CH-211-12d10) in which 11.5 kb of the *arg2* promoter drives GFP expression, to generate two transgenic line alleles with the same expression [*TgBAC(arg2:eGFP)sh571* and *TgBAC(arg2:eGFP)sh572*]. Owing to the higher fecundity in the *sh571* line, this line was used in the following studies (hereon termed the *arg2:GFP* line).

**Fig. 1.**
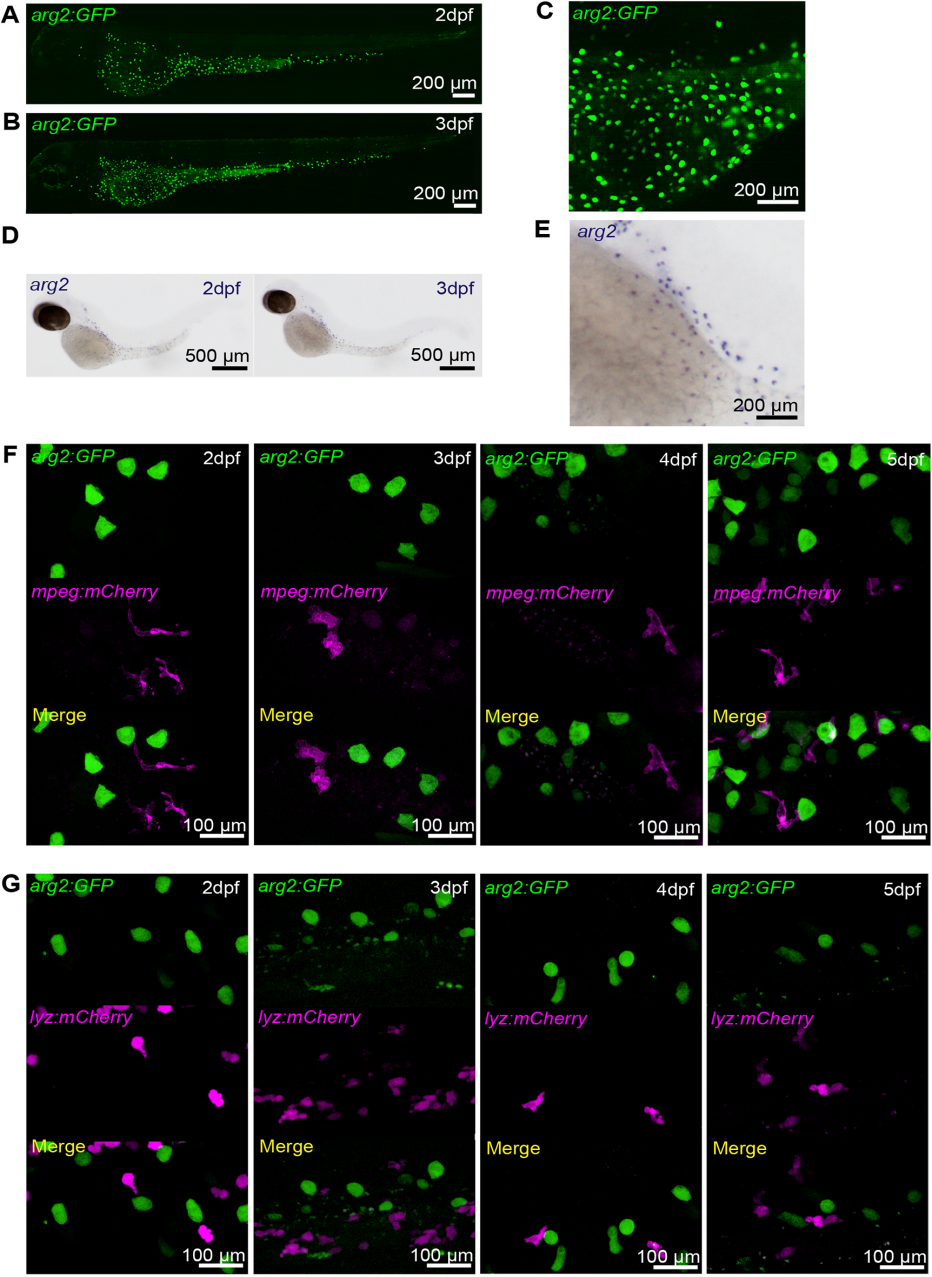
**The *TgBAC(arg2:eGFP)sh571* line shows GFP expression in ionocytes but not in resting macrophages and neutrophils, recapitulating the *arg2 in situ* hybridisation expression pattern.** (A,B) Light-sheet microscopy stereo micrographs of the *TgBAC(arg2:eGFP)sh571* (*arg2:GFP*) line shows ionocyte-specific expression at 2 dpf (A) and 3 dpf (B). (C) Enlarged image of the section over the yolk of B. (D) *arg2 in situ* hybridisation shows expression in cells over the yolk known as ionocytes at 2 and 3 dpf in unchallenged zebrafish (*n*=15/15 larvae accumulation over three independent experiments). (E) Enlarged image of the section over the yolk of 3 dpf larvae in D. (F) Stereo fluorescence micrograph of the *arg2:GFP* line crossed to the *Tg(mpeg1:mCherry)sh378* (*mpeg:mCherry*) line at 2, 3, 4 and 5 dpf, showing no overlap of *arg2:GFP* expression in ionocytes with *mpeg:mCherry*-positive (magenta) macrophages. Sixty larvae in total were screened for macrophage-specific *arg2:GFP* expression over three independent experiments. (G) Stereo fluorescence micrograph of the *arg2:GFP* line crossed to the *Tg(lyz:nfsB.mCherry)sh260* (*lyz:mCherry*) line at 2, 3, 4 and 5 dpf, showing no overlap of *arg2:GFP* expression in ionocytes with *lyz:mCherry*-positive (magenta) neutrophils. Sixty larvae in total were screened for neutrophil *arg2:GFP* expression over three independent experiments.

In unchallenged *arg2:GFP* larvae, the transgene was expressed in cuboidal cells in the skin, distributed over the yolk and caudal vein regions at 2 dpf (Fig. 1A) and 3 dpf (Fig. 1B,C). A subset of ionocytes, cells in the skin responsible for the transport of sodium ions, also known as H^+^-ATPase-rich cells (HRCs), have been shown to express high levels of *arg2* by *in situ* hybridisation in zebrafish (Eisenhoffer et al., 2017; Hsiao et al., 2007; Jänicke et al., 2007). *arg2:GFP* expression recapitulated the *arg2 in situ* hybridisation pattern, labelling ionocytes (Fig. 1D,E). To determine whether any of the cells over the yolk area expressing *arg2:GFP* were leukocytes, we crossed the *arg2:GFP* line with a macrophage transgenic line [*Tg(mpeg1:mCherry)sh378*, hereon termed *mpeg:mCherry*] and a neutrophil transgenic line [*Tg(lyz:nsfB.mCherry)sh260*, hereon termed *lyz:mCherry*]. Under resting conditions, there was no overlap between *mpeg:mCherry*-positive macrophages and *arg2:GFP-*positive cells (Fig. 1F) or between *lyz:mCherry-*positive neutrophils and *arg2:GFP-*positive cells (Fig. 1G) at 2, 3, 4 or 5 dpf. These data indicate that *arg2:GFP* is not expressed at detectable levels in immune cells in resting conditions at these developmental timepoints, matching *in situ* hybridisation data.

### *arg2:GFP* is predominantly upregulated by neutrophils after tailfin transection

**Fig. 2.**
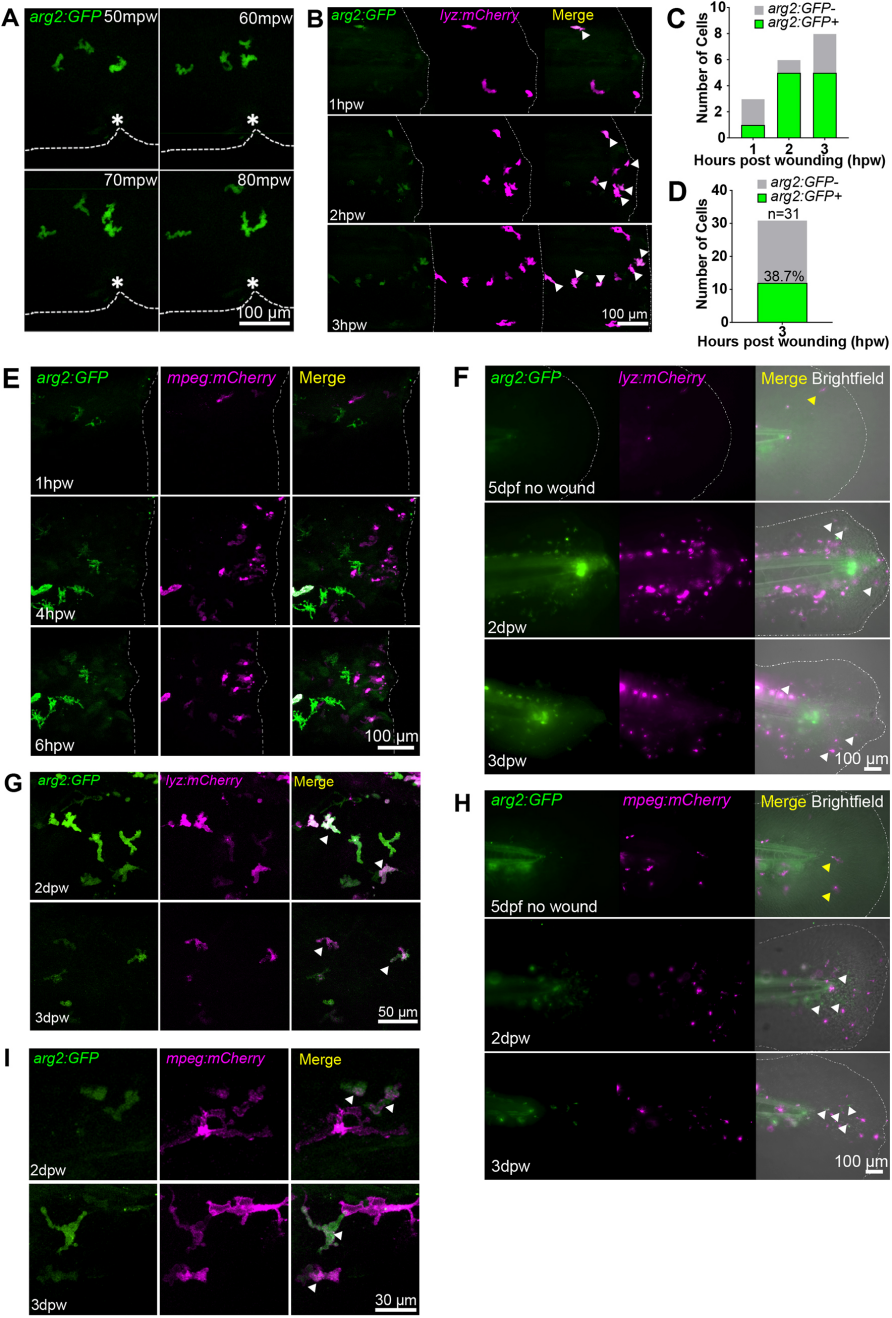
**Neutrophils express *arg2:GFP* after wound challenge.** (A) Fluorescence confocal timelapse micrographs of *arg2:GFP*-positive cells migrating towards a tailfin nick wound performed at 3 dpf at early timepoints post injury. Dashed lines indicate the edge of the fin and the asterisks mark the nick wound. mpw, minutes post wound. (B) Fluorescence confocal micrographs of the *arg2:GFP* line crossed to the *lyz:mCherry* line showing overlap of *arg2:GFP* with neutrophils at a tailfin wound performed at 3 dpf (dashed-dotted lines). Arrowheads indicate *arg2:GFP*-positive neutrophils migrating at the wound. Example timelapse images from two independent experiments with six larvae were imaged. (C) Quantification of *arg2:GFP*-positive neutrophils from B. (D) Number of neutrophils at the wound site that were *arg2:GFP* positive at 3 hpw. *n*=6 larvae combined from two independent experiments. (E) Fluorescence confocal timelapse micrographs of the *arg2:GFP* line crossed to the *mpeg:mCherry* line showing no overlap of macrophages with *arg2:GFP* expression early after injury performed at 3 dpf (dashed-dotted lines indicate the wound). The high exposure of the green channel to detect the earliest signs of *arg2:GFP* expression means that the autofluorescence of pigment cells is also evident, but these do not colocalise with *mpeg:mCherry*-positive cells. Sixty larvae in total were screened for macrophage *arg2:GFP* expression over three independent experiments. (F) Fluorescence widefield micrographs of the *arg2:GFP* line crossed to the *lyz:mCherry* line. The upper panels show an uninjured tailfin with few neutrophils (yellow arrowhead) and no immune cell-specific *arg2:GFP* expression overlap at 5 dpf. The middle panels show an injured tailfin (injury performed at 2 dpf) at 2 dpw (4 dpf) showing overlap between *lyz:mCherry* and *arg2:GFP* expression (white arrowheads). The lower panels show an injured tailfin at 3 dpw (5 dpf) showing overlap between *lyz:mCherry* and *arg2:GFP* expression (white arrowheads). The dashed-dotted lines indicate the edge of the tailfin fold. (G) Fluorescence confocal micrographs of the *arg2:GFP* line crossed to the *lyz:mCherry* line at 2 dpw (upper panels) and 3 dpw (lower panels), showing examples of *lyz:mCherry-*positive *arg2:GFP-*expressing cells (arrowheads) in the proximity of the healing tailfin wound performed at 2 dpf. (H) Fluorescence widefield micrographs of the *arg2:GFP* line crossed to the *mpeg:mCherry* line. The upper panels show an uninjured tailfin with few macrophages (yellow arrowheads) and no immune cell-specific *arg2:GFP* expression at 5 dpf. The middle panels show an injured tailfin (injury performed at 2 dpf) at 2 dpw (4 dpf) showing no overlap between *mpeg:mCherry* and *arg2:GFP* expression. *mpeg:mCherry*-negative cells expressing *arg2:GFP* with an amoeboid immune cell shape are shown by white arrowheads. The lower panels show an injured tailfin at 3 dpw (5 dpf) showing no overlap between *mpeg:mCherry* and *arg2:GFP* expression, with *mpeg:mCherry*-negative cells expressing *arg2:GFP* with an amoeboid immune cell shape shown by white arrowheads. The dashed-dotted lines indicate the edge of the tailfin fold. (I) Fluorescence confocal micrographs of the *arg2:GFP* line crossed to the *mpeg:mCherry* line at 2 dpw (upper panels) and 3 dpw (lower panels), showing examples of *mpeg:mCherry-*positive *arg2:GFP-*expressing cells in the proximity of the healing tailfin wound (injury performed at 2 dpf) (arrowheads).

To assess whether *arg2:GFP* expression is upregulated in innate immune cells during an immune response, we challenged 2 dpf larvae with a sterile tailfin wound. Using a tailfin nick model, we identified that highly mobile immune cells migrating towards the wound expressed *arg2:GFP* within the first hour of timelapse microscopy (Fig. 2A). We reasoned that these cells were neutrophils, owing to their size and amoeboid shape alongside their rapid migration towards the wound. To investigate whether neutrophils expressed *arg2:GFP* after injury, we crossed the *arg2:GFP* line with the neutrophil *lyz:mCherry* transgenic line. Timelapse microscopy demonstrated that a subpopulation of 39% of neutrophils arriving at the tailfin-transection wound were *arg2:GFP* positive by 3 h post wound (hpw) (Fig. 2B-D), with *arg2:GFP-*positive neutrophils at the site of injury present during the recruitment phase of inflammation (1-3 hpw), whereas expression was not observable in neutrophils away from the wound. There was no expression of *arg2:GFP* observed in *mpeg:mCherry*-positive macrophages in the recruitment phase of inflammation between 1 and 6 hpw (Fig. 2E).

After the first 24-36 h of inflammation, neutrophils have been largely cleared from the tailfin to allow for tailfin regeneration (Renshaw et al., 2006). We therefore assessed the *arg2:GFP* expression status of macrophages and neutrophils at regenerative stages between 2 and 3 days post wound (dpw). *arg2:GFP-*positive neutrophils were observed in the regenerating tailfin at 2 and 3 dpw, whereas in uninjured larvae, few neutrophils were present and they were *arg2:GFP* negative (Fig. 2F,G). In order to observe potential anti-inflammatory macrophages during the tailfin regeneration stage, *mpeg:mCherry* larvae crossed into the *arg2:GFP* line were imaged at the 2-3 dpw timepoints, by which time the fin has partially regenerated. In uninjured larvae, there were few *mpeg:mCherry-*positive macrophages in the tailfin fold at 5 dpf and these did not express *arg2:GFP* (Fig. 2H, top panel). In tailfin-transected larvae, there were increased numbers of macrophages in the 2 and 3 dpw healing/regenerating tailfin fold, but the majority of these were *arg2:GFP* negative, whereas *mpeg:mCherry-*negative cells with the morphology of neutrophils did express *arg2:GFP* (Fig. 2H, bottom two panels). Upon closer investigation using confocal microscopy, *mpeg:mCherry-*positive cells expressing *arg2:GFP* were identified at the wound at 2 and 3 dpw (Fig. 2I), although these were outnumbered by *arg2:GFP-*negative macrophages. Taken together, these data hint at the presence of *arg2*-expressing anti-inflammatory macrophages during tailfin regeneration.

### Infection challenge upregulates neutrophil *arg2:GFP* expression

**Fig. 3.**
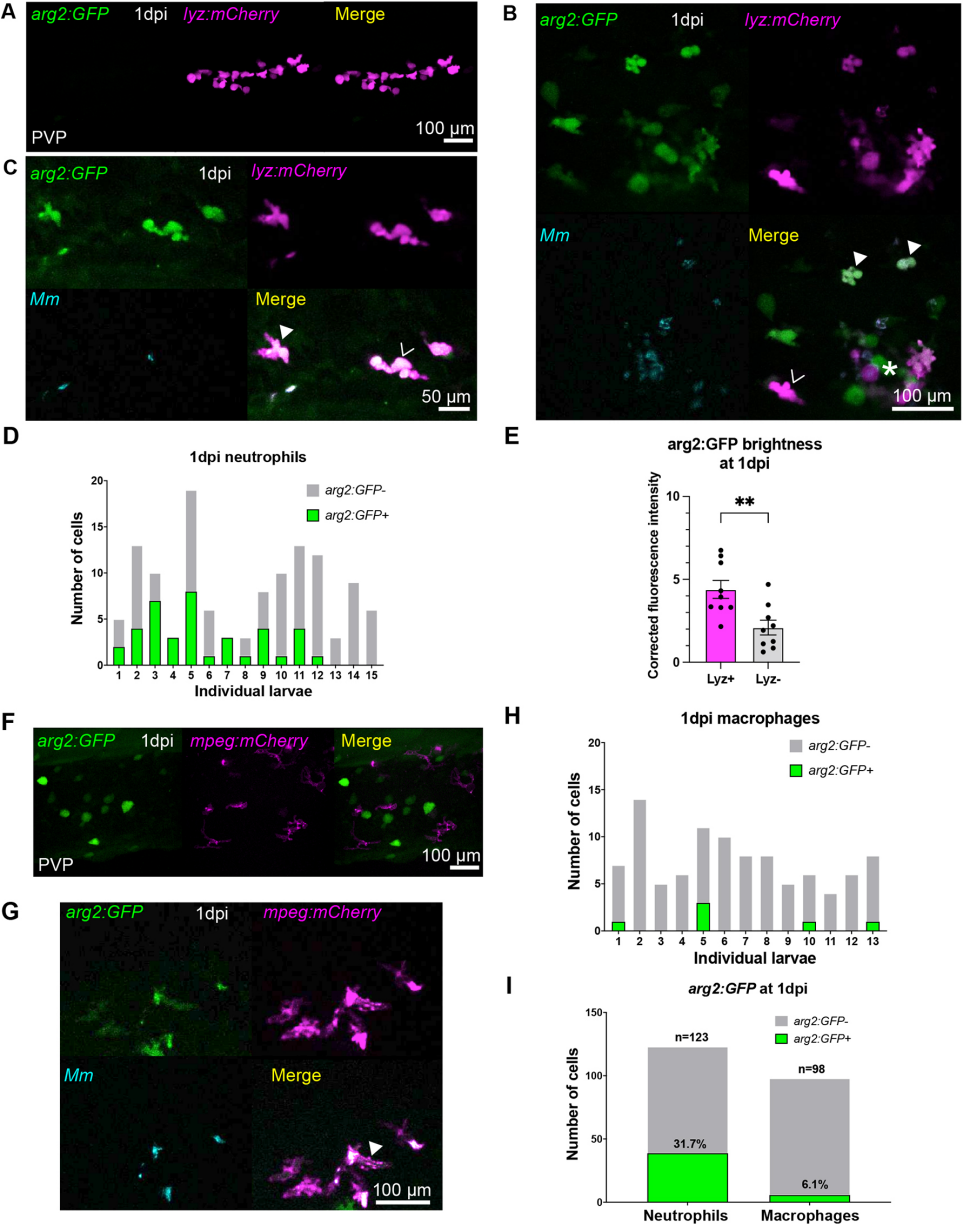
**Neutrophils are the predominant immune cell that express *arg2:GFP* post Mm challenge.** (A) Fluorescence confocal micrographs of 1 dpi (2 dpf) embryos (*arg2:GFP* line crossed to the *lyz:mCherry* line) after PVP mock infection at 1 dpf. (B) Fluorescence confocal micrographs of 1 dpi (2 dpf) embryos (*arg2:GFP* line crossed to the *lyz:mCherry* line) after Mm infection at 1 dpf showing GFP-positive neutrophils (filled arrowheads) and GFP-negative neutrophils (hollow arrowhead) around an area of high infection (asterisk). (C) Fluorescence confocal micrographs of 1 dpi (2 dpf) embryos (*arg2:GFP* line crossed to the *lyz:mCherry* line after Mm infection at 1 dpf showing that both infected (filled arrowhead) and non-infected (hollow arrowhead) neutrophils can express *arg2:GFP*. *n*=15 larvae imaged over three independent experiments. (D) Graph showing the number of *arg2:GFP*-positive and -negative neutrophils in a 40× region of interest in the caudal vein region that contained Mm bacteria, post infection, in individual larvae. Data shown are from *n*=15 larvae accumulated from three independent experiments. (E) Corrected fluorescence intensity of *arg2:GFP* expression in *lyz:mCherry*-positive neutrophils compared to that of cells with an immune morphology that were *lyz:mCherry* negative at 1 dpi (2 dpf). Data shown are from *n*=9 larvae accumulated from three independent experiments. *P*-values were calculated using unpaired two-tailed *t*-test. **P<0.01. (F) Fluorescence confocal micrographs of 1 dpi (2 dpf) embryos (*arg2:GFP* line crossed to the *mpeg:mCherry* line) after PVP control injection at 1 dpf. (G) Fluorescence confocal micrographs of 1 dpi (2 dpf) embryos (*arg2:GFP* line crossed to *mpeg:mCherry* line) after Mm infection at 1 dpf showing a single GFP-positive macrophage (filled arrowhead) in this field of view (representing one of six instances observed). (H) Graph showing the number of *arg2:GFP*-positive and -negative macrophages in a 40× region of interest in the caudal vein region that contained Mm bacteria post infection in individual larvae. (I) Graph showing the percentage of *arg2:GFP-*positive and -negative neutrophils and macrophages in a 40× region of interest around the infected caudal vein region. Data shown are from *n*=98-123 cells accumulated from 15 larvae for neutrophils and 13 larvae for macrophages over three independent experiments.

To investigate the expression of *arg2* after bacterial infection, *arg2:GFP* embryos were injected with *M. marinum* (Mm) at 1 dpf and imaged at 1 day post injection (dpi) (2 dpf). Neutrophil *arg2:GFP* expression was not observed in mock-infected (injected with 2% polyvinylpyrrolidone 40, referred to as PVP) controls (Fig. 3A), with ionocyte-specific expression used to confirm *arg2:GFP*-positive larvae. We assessed the early response to Mm infection at 1 dpi and found that a subpopulation of neutrophils express *arg2:GFP* early in infection (Fig. 3B). *arg2:GFP*-positive neutrophils were present in the vicinity of Mm infection (Fig. 3B), with both infected and uninfected neutrophils expressing *arg2:GFP* (Fig. 3C). A subset of neutrophils had *arg2:GFP* expression, with 31.7% (of *n*=123 neutrophils) of *lyz:mCherry*-positive neutrophils in the region of infection expressing GFP, suggestive of differential immune responses between individual neutrophils (Fig. 3D,I). *lyz:mCherry*-positive cells expressed *arg2:GFP* to a higher level than *lyz:mCherry*-negative cells with an immune cell morphology (potential macrophages) in the same larvae (Fig. 3E). At the 1 dpi (2 dpf) timepoint, macrophage *arg2:GFP* expression was also not observed in mock-infected (PVP) controls (Fig. 3F), with ionocyte-specific expression used to confirm *arg2:GFP*-positive larvae. Only 6.1% (of *n*=98 macrophages) of *mpeg:mCherry-*positive macrophages were positive for *arg2:GFP* in Mm-infected individuals (Fig. 3G-I).

**Fig. 4.**
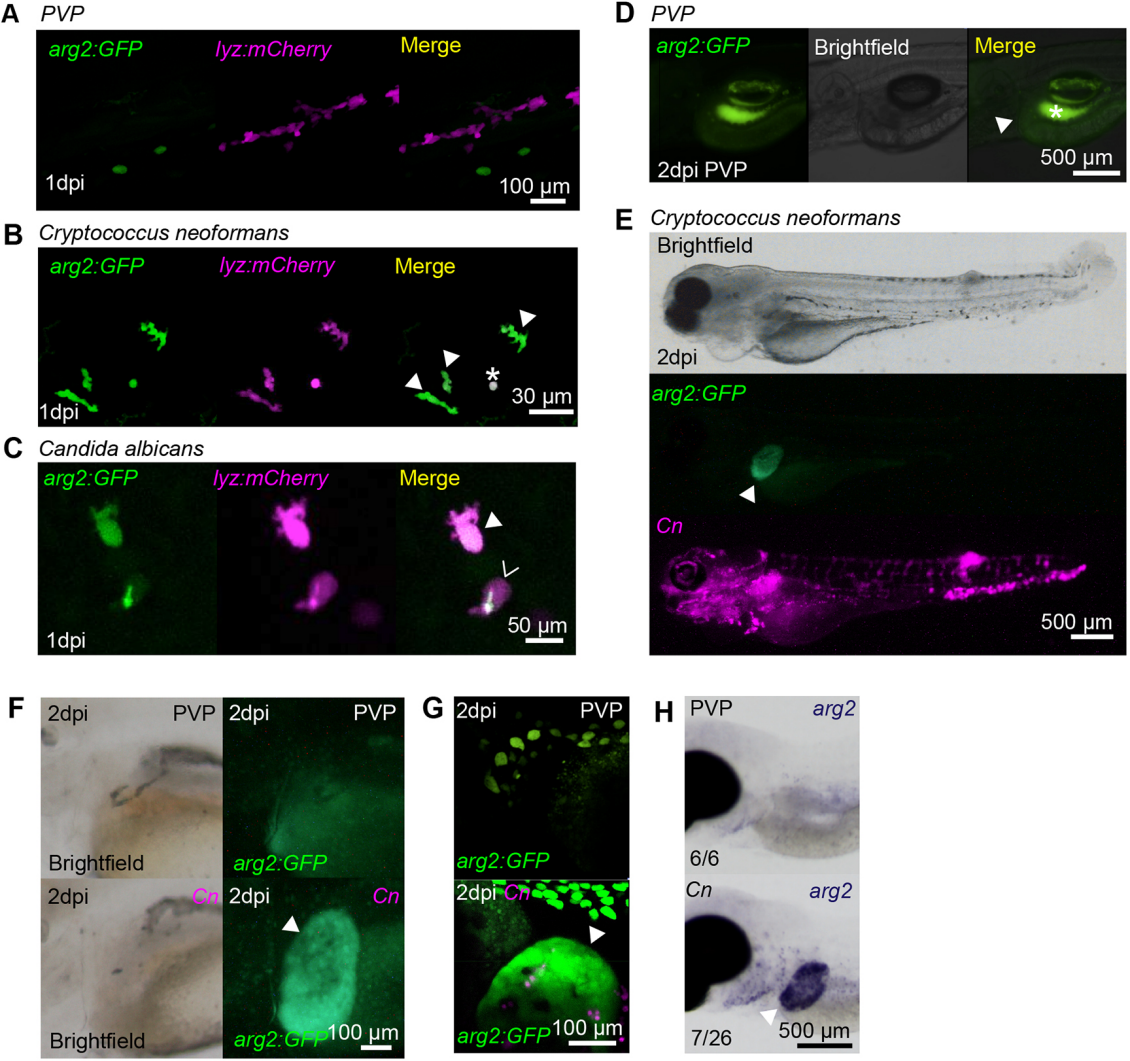
**Fungal infections upregulate *arg2:GFP* expression in neutrophils and the liver.** (A) Fluorescence confocal micrographs of 2 dpf embryos (*arg2:GFP* line crossed to the *lyz:mCherry* line) after PVP mock infection at 1 dpi showing no overlap between GFP and *lyz:mCherry* expression. (B) Fluorescence confocal micrographs of 1 dpi (2 dpf) embryos (*arg2:GFP* line crossed to the *lyz:mCherry* line) after *Cryptococcus neoformans* infection at 1 dpf showing GFP-positive neutrophils (filled arrowheads). The asterisk indicates a *Cryptococcus* that has autofluorescence in both channels. (C) Fluorescence confocal micrographs of 1 dpi (2 dpf) embryos (*arg2:GFP* line crossed to the *lyz:mCherry* line) after *Candida albicans* infection at 1 dpf showing a GFP-positive neutrophil (filled arrowhead) and a GFP-negative neutrophil (hollow arrowhead (the green fluorescence signal in this cell is autofluorescence from *Candida*, which in this instance has survived and formed a hypha). (D) Brightfield and fluorescence micrographs of *arg2:GFP* larvae at 2 dpi (3 dpf) after PVP injection at 1 dpf. The position of the *arg2:GFP-*negative liver is shown by the arrowhead. The contrast of the green fluorescence channel has been turned up sufficiently to show gut fluorescence (asterisk), in order to show that the liver is *arg2:GFP* negative. (E) Brightfield and fluorescence micrographs of 2 dpi (3 dpf) *arg2:GFP* larvae after *Cryptococcus neoformans* (Cn) infection at 1 dpf showing *arg2:GFP* liver-specific expression in an individual with high levels of infection. (F) Brightfield and widefield fluorescence micrographs of 2 dpi (3 dpf) *arg2:GFP* larvae after PVP mock infection or Cn infection at 1 dpf, showing *arg2:GFP* liver-specific expression (arrowhead) in the Cn-infected individual. (G) Fluorescence confocal micrographs of 2 dpi (3 dpf) *arg2:GFP* larvae after PVP mock infection or Cn infection at 1 dpf showing *arg2:GFP* liver-specific expression (arrowhead) in the Cn-infected individual (arrowhead). (H) Brightfield stereo micrographs of 2 dpi (3 dpf) embryos after PVP or Cn infection at 1 dpf and *arg2* whole-mount *in situ* hybridisation at 3 dpf showing *arg2* liver-specific expression (arrowhead) in an infected individual, not present in the PVP-injected larvae. Liver-specific expression of *arg2* was observed in *n*=7/26 Cn-infected larvae performed over three independent experiments.

Fungal infections have been shown to modulate host arginine metabolism via arginase (Wagener et al., 2017); therefore, we assessed *arg2:GFP* expression in two well-characterised fungal zebrafish infection models – *C. albicans* (Brothers and Wheeler, 2012) and *C. neoformans* (Bojarczuk et al., 2016). Mock infection with PVP caused no neutrophil *arg2:GFP* expression (Fig. 4A). In both fungal infections, *arg2:GFP* was observed in a subpopulation of *lyz:mCherry-*positive neutrophils at 1 dpi (Fig. 4B,C). As with Mm infection, subsets of neutrophils both with or without internalised pathogen were *arg2:GFP* positive in *C. albicans* infection (Fig. 4C). *arg2:GFP-*positive *mpeg:mCherry* macrophages were also observed in *C. neoformans* infection at 1 dpi; however, these were outnumbered by *arg2:GFP*-negative macrophages and only three examples were identified (Fig. S2). In cryptococcal-infected larvae with a high fungal burden, *arg2:GFP* expression was observed in the liver (Fig. 4E) at timepoints when *arg2:GFP* expression was not present in PVP-injected larvae (Fig. 4D). This was especially apparent in *Cryptococcus* infections at 2 dpi (Fig. 4F,G) and was confirmed by *in situ* hybridisation (Fig. 4H). Arginase is a well-characterised liver enzyme (Wright et al., 2004), yet in unchallenged embryos (Fig. 1A-E) or PVP-injected larvae (Fig. 4D), there was no visible liver expression of *arg2:GFP*. Tissue-restricted *arg2:GFP* expression in the liver area was also observed in examples of heavily infected Mm larvae (Fig. S3). Like cryptococcal-infected larvae, liver expression occurred in individuals highly infected with Mm, but was not observed until later after infection (at 4 dpi), potentially reflecting the slower doubling time/pathogenesis of Mm compared to *Cryptococcus*.

### *arg2:GFP* is expressed in cells associated with developing granulomas

**Fig. 5.**
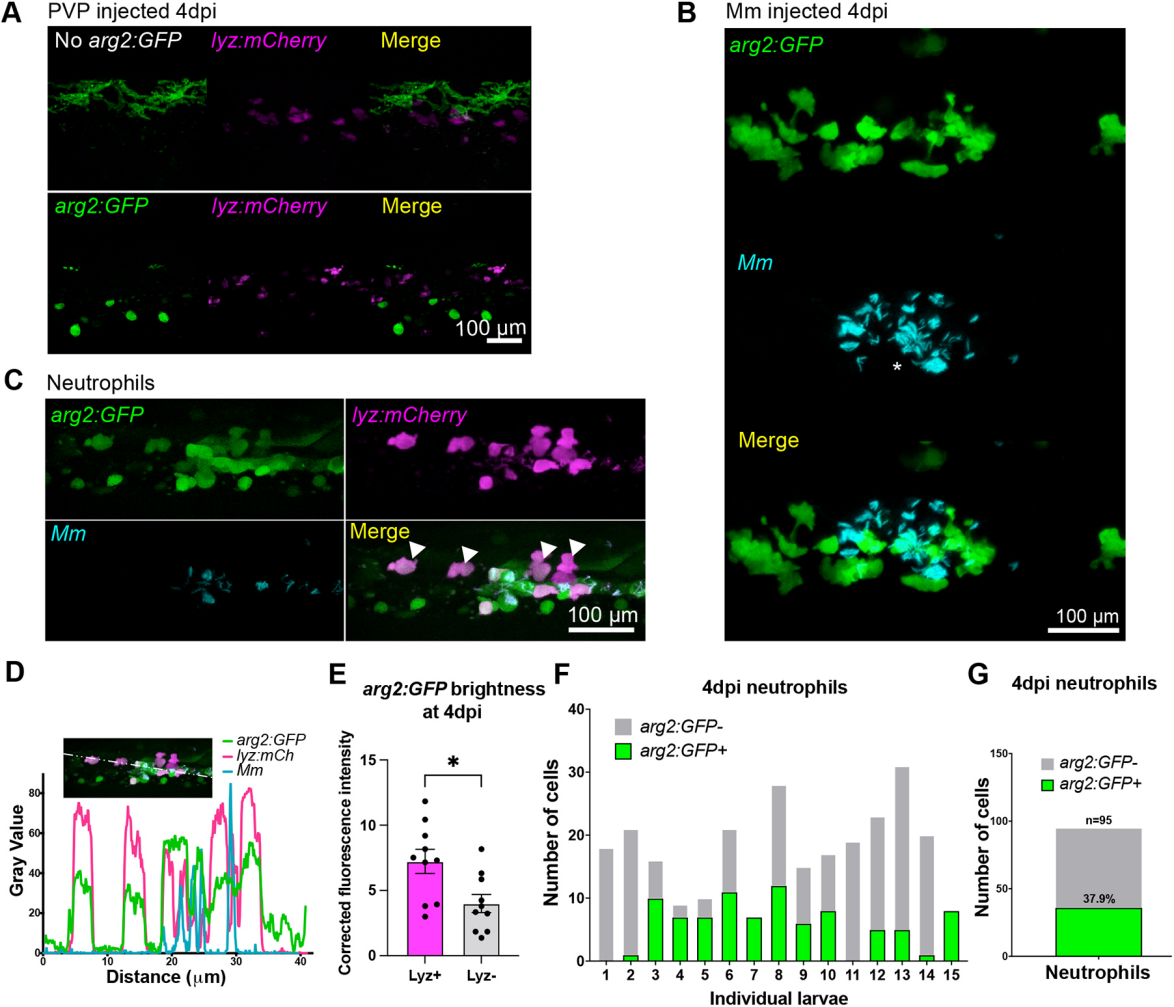
**Granuloma-associated neutrophils express *arg2:GFP.*** (A) Fluorescence confocal micrographs of 4 dpi (5 dpf) embryos [*lyz:mCherry* line crossed to the wild-type line (no *arg2:GFP*) or *arg2:GFP* line] after PVP control injection at 1 dpf. Only pigment autofluorescence and ionocyte-specific expression of *arg2:GFP* is present. (B) Fluorescence confocal micrographs of 4 dpi (5 dpf) *arg2:GFP* embryos after Mm infection at 1 dpf showing granuloma-associated *arg2:GFP*-positive cells. (C) Fluorescence confocal micrographs of 4 dpi (5 dpf) embryos (*arg2:GFP* line crossed to the *lyz:mCherry* line) after Mm infection at 1 dpf, showing neutrophils positive for *arg2:GFP* (filled arrowheads). (D) Line analysis of a cross section through a granuloma showing fluorescence values of *arg2:GFP*, *lyz:mCherry* and Mm. (E) Corrected fluorescence intensity of *arg2:GFP* in *lyz:mCherry*-positive neutrophils compared to that in cells with immune morphology that were *lyz:mCherry* negative at 4 dpi (5 dpf). Data shown are from *n*=10 larvae accumulated from three independent experiments. The *P*-value was calculated using unpaired two-tailed *t*-test. *P<0.05. (F) Graph showing the number of *arg2:GFP*-positive and -negative neutrophils in a 40× region of interest in the caudal vein region that contained Mm bacteria, at 4 dpi (5 dpf), in individual larvae. Data shown are from *n*=15 larvae accumulated from three independent experiments. (G) Graph showing the percentage of *arg2:GFP-*positive and -negative granuloma-associated neutrophils at 4 dpi (5 dpf). Data shown are from *n*=95 cells from 15 larvae accumulated over three independent experiments.

*arg2:GFP* expression was assessed at a later stage of Mm infection (4 dpi), a stage at which innate immune granulomas are forming and when it is likely that leukocyte phenotypes are more diverse and polarised owing to immune modulation by mycobacteria (Cronan et al., 2021). PVP-control-injected larvae had minimal *arg2:GFP* expression, apart from ionocyte-specific expression and background green signal from the autofluorescence of pigment cells (Fig. 5A). In Mm-infected larvae, *arg2:GFP* was expressed in cells associated with developing granulomas at 4 dpi (Fig. 5B). The *lyz:mCherry-*positive cells appeared to express higher levels of *arg2:GFP* than other granuloma-associated cells (Fig. 5C,D). When quantified, the *lyz:mCherry*-positive cells had greater *arg2:GFP* fluorescence than *lyz:mCherry*-negative cells with an immune cell morphology (potential macrophages) in the same larvae (Fig. 5E). *arg2:GFP-*positive granuloma-associated neutrophils represented 37.9% of neutrophils imaged around granulomas, with the remaining 62.1% having no detectable *arg2:GFP* expression (Fig. 5F,G).

**Fig. 6.**
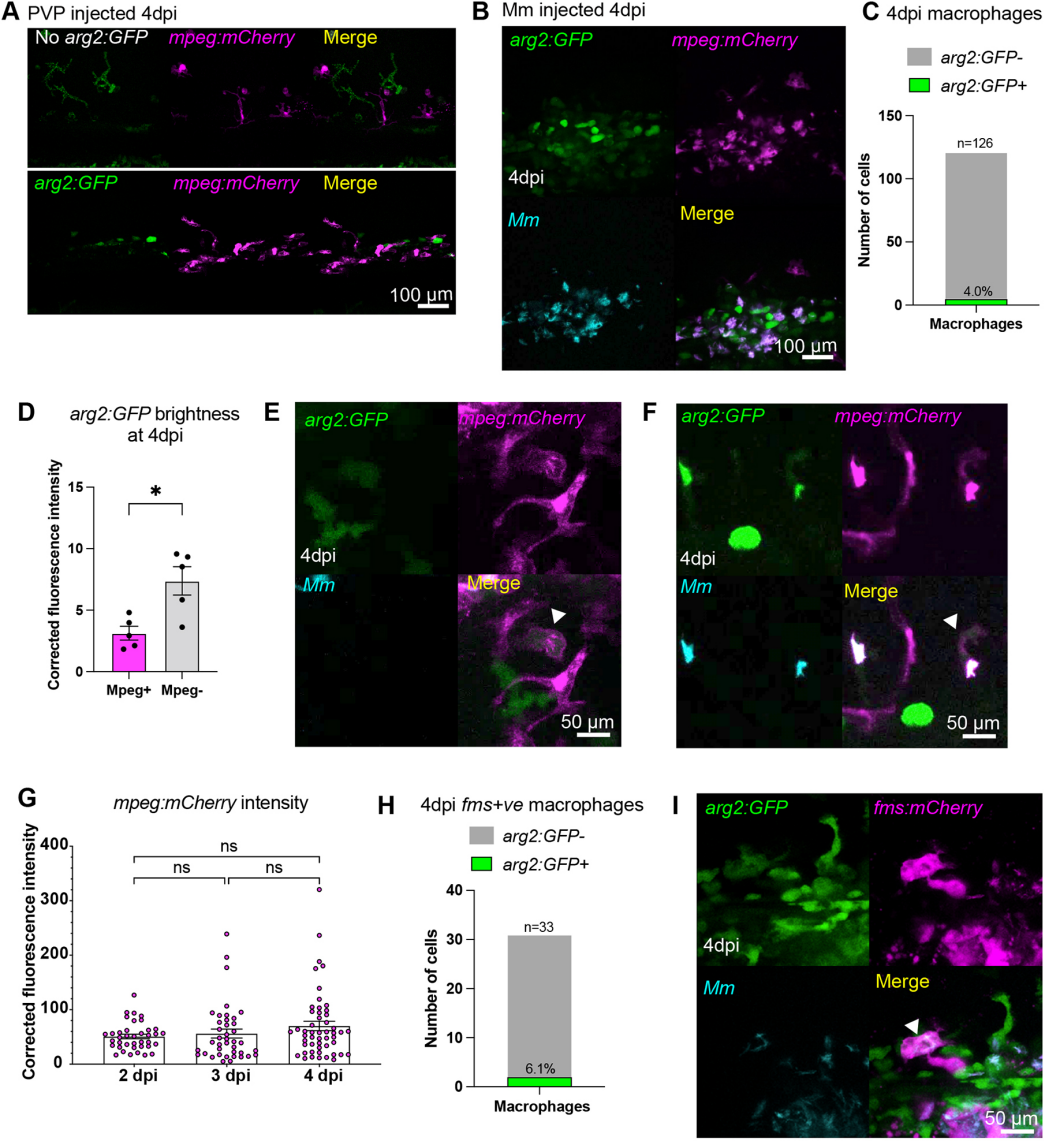
**A subset of granuloma-associated macrophages express *arg2:GFP.*** (A) Fluorescence confocal micrographs of 4 dpi (5 dpf) embryos [*mpeg:mCherry* line crossed to the wild-type line (no *arg2:GFP*) or *arg2:GFP* line] after PVP control injection at 1 dpf. Only pigment autofluorescence and ionocyte-specific expression of *arg2:GFP* is present. (B) Fluorescence confocal micrographs of 4 dpi (5 dpf) embryos (*arg2:GFP* line crossed to the *mpeg:mCherry* line) after Mm infection at 1 dpf. (C) Graphs showing the percentage of *arg2:GFP-*positive and -negative granuloma-associated macrophages at 4 dpi (5 dpf). Data shown are from *n*=126 cells from 15 larvae accumulated from three independent experiments. (D) Corrected fluorescence intensity of *arg2:GFP* in *mpeg:mCherry*-positive macrophages compared to that in cells with immune morphology that were *mpeg:mCherry* negative at 4 dpi (5 dpf). Data shown are from *n*=12 larvae accumulated from three independent experiments. The *P*-value was calculated using unpaired two-tailed *t*-test. *P<0.05. (E) Fluorescence confocal micrographs of 4 dpi (5 dpf) embryos (*arg2:GFP* line crossed to the *mpeg:mCherry* line) after Mm infection at 1 dpf showing a non-infected, *arg2:GFP*-positive macrophage (arrowhead). (F) Fluorescence confocal micrographs of 4 dpi (5 dpf) embryos (*arg2:GFP* line crossed to the *mpeg:mCherry* line) after Mm infection at 1 dpf showing an infected, *arg2:GFP*-positive macrophage (arrowhead). (G) Corrected fluorescence intensity of mCherry in *mpeg:mCherry-*positive macrophages at 2, 3 and 4 dpi (3, 4 and 5 dpf, respectively) after Mm infection at 1 dpf. Data shown are from *n*=10-12 larvae. The *P*-value calculated using a one-way ANOVA (with Bonferonni post-test adjustment). ns, not significant. (H) Graph showing the percentage of *arg2:GFP-*positive and -negative granuloma-associated macrophages marked with *Tg(fms:Gal4.VP16)i186;Tg(UAS:nfsB.mCherry)i149* (*fms:mCherry*) expression, at 4 dpi (5 dpf). Data shown are from *n*=33 cells accumulated from two independent experiments. (I) Fluorescence confocal micrographs of 4 dpi (5 dpf) embryos (*arg2:GFP* line crossed to the *fms:mCherry* line) after Mm infection at 1 dpf, showing an *arg2:GFP*-positive macrophage (arrowhead).

It was noted that there were also granuloma-associated *arg2:GFP-*expressing cells that were *lyz:mCherry* negative (Fig. 5C), some containing phagocytosed bacteria (Fig. 5C), and we hypothesised these to be macrophages. PVP-control-injected larvae had minimal *arg2:GFP* expression in macrophages, but did have ionocyte-specific expression and background green signal from the autofluorescence of pigment cells (Fig. 6A). There were few *mpeg:mCherry*-positive macrophages that were *arg2:GFP* positive at 4 dpi (4% of those imaged; Fig. 6B,C). At 4 dpi, *mpeg:mCherry*-positive macrophages had lower fluorescence levels of *arg2:GFP* compared to *mpeg:mCherry*-negative/*arg2:GFP-*positive granuloma-associated cells that would include neutrophils (Fig. 6D). As was the case with neutrophils, *arg2:GFP* expression was observed in both infected and non-infected *mpeg:mCherry-*positive macrophages around granulomas (Fig. 6E,F). The low number of *mpeg:mCherry/arg2:GFP* double-positive macrophages observed at 4 dpi, alongside *arg2:GFP*-positive neutrophils, did not appear to account for the many granuloma-associated *arg2:GFP*-positive cells that were observed, with many appearing to be *mpeg:mCherry* negative (Fig. 6B). As it has been previously shown that the *mpeg* promoter is downregulated by Mm at 4 dpi (Benard et al., 2015), the fluorescence brightness of *mpeg:mCherry* expression was assessed at 2, 3 and 4 dpi (3, 4 and 5 dpf, respectively) after Mm infection at 1 dpf. There was no difference in red fluorescence between 2, 3 or 4 dpi, suggesting that putative Mm-infection-induced downregulation of the *mpeg* promoter did not impact the brightness of the macrophage transgenic fluorescence at 4 dpi (Fig. 6G). The number of *arg2:GFP*-positive macrophages at 4 dpi was assessed using a second macrophage transgenic line [*Tg(fms:Gal4.VP16)i186;Tg(UAS:nfsB.mCherry)i149*, hereafter referred to as *fms:mCherry* for clarity]. The proportion of *arg2:GFP*-positive *fms:mCherry* macrophages at 4 dpi was approximately equivalent to that observed in *mpeg:mCherry* macrophages (Fig. 6H,I).

Taken together, these data show that Mm granulomas have arginase*-*expressing neutrophils and that other granuloma-associated cells, including potential anti-inflammatory macrophages, also express arginase but to a lower level.

## DISCUSSION

Zebrafish transgenic lines have allowed *in vivo* exploration of the pro-inflammatory immune response (including *il1b* and *tnfa*) to a variety of stimuli; however, until now, there has been a lack of similar tools for anti-inflammatory factors (Marjoram et al., 2015; Nguyen-Chi et al., 2015; Ogryzko et al., 2019). Here, we describe a new transgenic line for *arg2* and show that this transgene is upregulated shortly after immune challenge in neutrophils and a small subset of macrophages. These data correlate with a growing body of evidence that suggest that anti-inflammatory factors are expressed early after immune challenge to suppress hyper-inflammation, before a switch to an anti-inflammatory environment is required at later stages for tissue healing and restoration of homeostasis (Cicchese et al., 2018).

The *arg2:GFP* line shows that neutrophils are the predominant immune cell type that express *arg2* early after immune challenge in zebrafish. The observation of *arg2* expression in zebrafish neutrophils fits with our previous observations that zebrafish neutrophils are the primary innate immune cell that produce antimicrobial RNS after Mm infection and suggests a neutrophil iNOS/arginase balance (Elks et al., 2013). Human neutrophils also produce high levels of RNS (Munder et al., 2005), differing from expression in mice, in which it is primarily macrophages that produce RNS/arginase (Abebe et al., 2013; El Kasmi et al., 2008; Jacobsen et al., 2007; Munder et al., 2005). Human polymorphonuclear neutrophils produce high levels of arginase at transcript and protein levels following immune challenge and, in resting states, this may act as a negative regulator of the RNS response, as is the case in murine macrophages (Abebe et al., 2013; El Kasmi et al., 2008; Jacobsen et al., 2007; Munder et al., 2005).

Our analysis of the zebrafish *arg2:GFP* transgene and *in situ* hybridisation did not detect leukocyte *arg2* expression in resting conditions; however, *arg2* expression was detected by RNAseq and RT-qPCR of FACS-purified leukocyte populations. This suggests that either the transgenic and *in situ* hybridisation techniques were not as sensitive as RNAseq or that techniques to purify immune cells in RNAseq studies have led to upregulation of *arg2* not present in the intact zebrafish. FACS-purified neutrophils expressed higher levels of *arg2* compared to its levels in sorted macrophages and the background of the larvae, matching our observations in the *arg2:GFP* line once activated by immune challenge. In the original publication of these FACS-purified populations (Nguyen-Chi et al., 2014), it was noted that immune cells may have been activated by the dissociation/FACS process, as elevated expression of proinflammatory cytokines such as *il1b* was observed, highlighting the technical challenges involved in *ex vivo* experimentation on leukocyte polarisation (Nguyen-Chi et al., 2014). FACS and subsequent RNAseq have been extensively and successfully used to identify and assess activation states of both macrophages and neutrophils; however, immune cells are removed from their local microenvironment during dissociation and careful technique development is required to avoid inadvertent activation (Nguyen-Chi et al., 2015; Rougeot et al., 2014, 2019). The addition of intact transgenic lines such as the *arg2:GFP* line, alongside transcriptomics requiring dissociation, will be powerful tools to further dissect immune cell polarisation.

The mechanisms balancing innate immune regulation during inflammatory and infection responses are not well understood *in vivo*. Neutrophil *arg2:GFP* expression was observed at timepoints that are considered to be pro-inflammatory stages of inflammation and infection. *arg2:GFP* expression was reminiscent of our previous observations using the pro-inflammatory *TgBAC(il-1β:GFP)sh445* transgenic line, in which neutrophil *il1b* was found at 1 hpw in the tailfin and 1 dpi in Mm infections, the same timepoints at which *arg2:GFP* expression was observed (Ogryzko et al., 2019). This suggests that anti-inflammatory arginase expression coincides with pro-inflammatory signals in neutrophils, providing evidence for a balanced response. The observation of immune cells producing anti-inflammatory signals upon immune challenge has been reported previously, but to date this has been mainly described in macrophages (Cicchese et al., 2018). It is becoming clear that a balanced response to immune challenge, including both pro- and anti-inflammatory signals, is beneficial in disease control (Cicchese et al., 2018). In murine macrophages, it has been demonstrated *in vitro* that ARG1 is produced soon after infection and can have immunomodulatory effects (El Kasmi et al., 2008). Our findings open up the possibility that a similar balance may exist in neutrophils and add to recent evidence suggesting that neutrophil phenotypes are more diverse and nuanced than previously appreciated (Ballesteros et al., 2020; Giese et al., 2019).

Some pathogens have evolved to disrupt the pro- and anti-inflammatory response, keeping pro-inflammatory factors low and increasing anti-inflammatory signals to allow for immune cell evasion and survival. One such pathogen is *Mycobacterium tuberculosis*, which suppresses an initial pro-inflammatory response, in part by upregulation of macrophage arginase, to allow for intra-phagocyte survival and intracellular growth and proliferation to form hallmark granuloma structures (El Kasmi et al., 2008). This makes arginase a potential target for therapeutic intervention during tuberculosis. In murine tuberculosis models, macrophage arginase expression is associated with decreased bacterial killing and is an immunomodulatory target of *M. tuberculosis* (El Kasmi et al., 2008). Similar observations have been described in fungal infections, with mice infected with *Cryptococcus* (*Cryptococcus gattii*) having elevated levels of arginase expression in lung tissues (Oliveira-Brito et al., 2020). In human-monocyte-derived macrophages, *Candida albicans* infection induces arginase expression, which blocks host nitric oxide (NO) production as a fungal survival mechanism via chitin exposure (Wagener et al., 2017). Our findings using the *arg2:GFP* line are consistent with these mammalian observations, with early arginase expression observed in innate immune cells after Mm and fungal infection. Further investigation is required to understand the molecular mechanisms of this intriguing host-pathogen interaction.

Arginase has been described as an anti-inflammatory macrophage marker due, in part, to high expression levels in experiments using anti-inflammatory stimuli such as IL-4 or IL-13 to drive monocytes towards M2/anti-inflammatory phenotypes, as well as observations in other murine macrophage models (Mantovani et al., 2004). We observed macrophages expressing *arg2:GFP* during infection and at wound-healing stages of tailfin transection. Our findings complement studies on a zebrafish pro-inflammatory macrophage *tnfa* transgenic line (Nguyen-Chi et al., 2014), which suggest that an anti-inflammatory population of macrophages exists based on the switching off of *tnfa:GFP* in some macrophages during the wound-healing phase of tailfin transection, and are consistent with anti-inflammatory macrophages being present in the developing zebrafish larvae (Nguyen-Chi et al., 2015). However, in both inflammation and infection, arginase-expressing macrophages were much less frequent than arginase-expressing neutrophils, and neutrophil arginase expression predominated. It is important to note that the *mpeg* promoter used to mark macrophages in our study is downregulated by Mm infection (Benard et al., 2015); therefore, it is possible that our observations using the *mpeg:mCherry* line is an underestimation of the population of macrophages that express *arg2:GFP* during Mm infection. The possibility of this downregulation is evident from our data as there are many *arg2:GFP-*positive granuloma-associated cells, some with phagocytosed bacteria, that expressed *arg2:GFP* but were neutrophil-marker negative, indicative of macrophages that lack a visible *mpeg:mCherry* marker. However, in our experiments, *mpeg:mCherry* fluorescence levels in the transgenic line were not decreased at 4 dpi, indicating that the *mpeg* promoter remained active or that any inactivation by Mm had not yet affected the mCherry levels. Furthermore, investigation using a separate macrophage marker, *fms:mCherry*, also showed few *arg2:GFP*-positive macrophages at 4 dpi, with numbers approximately equivalent to those observed in *mpeg:mCherry* larvae. The *fms:mCherry* line used was not a direct promoter driver, but uses the *gal4:uas* system, which is silenced in zebrafish over generations, so there remains the possibility that not all macrophages are labelled in these larvae (Akitake et al., 2011). The granuloma-associated *arg2:GFP* expression observed may also be from epithelioid-like cells that make up a large proportion of the zebrafish Mm granuloma structure, some of which are macrophage derived but may have lost macrophage markers (Cronan et al., 2021). Here, we have identified *mpeg:mCherry*-positive granuloma-associated macrophages that express *arg2*; however, it remains unclear as to exactly how many macrophages are polarised towards this potential anti-inflammatory phenotype in zebrafish. Characterising the macrophage polarisation infection response fully will require further study, for which the *arg2:GFP* zebrafish line will be an important tool.

Outside of immune cells, the *arg2:GFP* line has also illuminated arginase expression in the liver in highly infected individuals and in ionocytes, during both resting and inflammatory states. Arginase is a well-characterised liver enzyme (Berüter et al., 1978; Haraguchi et al., 1987); therefore, expression in the liver was not unexpected, although the function of the upregulated liver-specific expression in highly infected individuals remains unclear. Interestingly, ionocytes have recently been described as a new airway epithelial cell type in humans and mice and it is these cells that most highly express cystic fibrosis transmembrane conductance regulator (CFTR), the anion channel that is mutated in cystic fibrosis patients (Shah et al., 2022). The *arg:GFP* line is a new tool that could be used to investigate the roles of these intriguing cells *in vivo*.

Our data indicate that *arg2*, an important anti-inflammatory mediator, is produced early after immune challenge, predominantly by neutrophils. The *arg2:GFP* line is an exciting addition to the zebrafish transgenic toolbox with which to investigate innate immunity during infections. It has the potential to be applied to multiple zebrafish disease models of infection and inflammation and may also be relevant to any models with an inflammatory component, from ageing to cancer.

## MATERIALS AND METHODS

### Ethics statement

Animal work was carried out according to guidelines and legislation set out in UK law in the Animals (Scientific Procedures) Act 1986, under Project License P1A4A7A5E or PP7684817. Ethical approval was granted by the University of Sheffield Local Ethical Review Panel.

### Fish husbandry

All zebrafish were raised in the Biological Services Unit (BSU) aquarium (University of Sheffield, UK) and maintained according to standard protocols (https://zfin.org/) in Home Office-approved facilities. Adult fish were maintained at 28°C with a 14 h/10 h light/dark cycle.

To investigate expression in immune cells, the *TgBAC(arg2:eGFP)sh571* (*arg2:GFP*) reporter line was crossed with the macrophage reporters *Tg(mpeg1:mCherryCAAX)sh378* (Bojarczuk et al., 2016) (*mpeg:mCherry*) and *Tg(fms:Gal4.VP16)i186;Tg(UAS:nfsB.mCherry)i149* (Gray et al., 2011) (*fms:mCherry*), and the neutrophil reporter *Tg(lyz:nfsB.mCherry)sh260* (Buchan et al., 2019) (*lyz:mCherry*) to generate embryos for experiments.

### Generation of *arg2:GFP* transgenic zebrafish

An eGFP SV40 polyadenylation cassette was inserted at the *arg2* ATG start site of the zebrafish BAC CH-211-12d10 using established protocols (Renshaw et al., 2006). Inverted *Tol2* elements were inserted into the chloramphenicol coding sequence and the resulting modified BAC containing 115,130 bp of the *arg2* promoter region was used. We identified two founder zebrafish (allele codes sh571 and sh572) and raised colonies. The embryos of both alleles had the same GFP expression pattern. The data generated in this manuscript is from the *TgBAC(arg2:eGFP)sh571* transgenic line (*arg2:GFP*) as this line had a higher fecundity than *TgBAC(arg2:eGFP)sh572*. The *TgBAC(arg2:eGFP)sh571* strain can be requested by contacting the corresponding author.

### Tailfin transection

To induce an inflammatory stimulus, 2- or 3-dpf zebrafish were anaesthetised in 0.168 mg/ml tricaine (MS-222, Sigma-Aldrich) in E3 medium and visualised under a dissecting microscope. Using a scalpel blade (5 mm depth, World Precision Instruments) the tailfin was transected after the circulatory loop as previously described, while ensuring that the circulation remained intact (Elks et al., 2011a).

### Pathogen strains and culture

Bacterial infection experiments were performed using *Mycobacterium marinum* strain M (American Type Culture Collection #BAA-535) containing the pSMT3-Crimson vector, with liquid cultures prepared from bacterial plates (van der Sar et al., 2009). Liquid cultures were washed and prepared in 2% polyvinylpyrrolidone 40 (PVP40) solution (Sigma-Aldrich) as previously described for injection (Benard et al., 2012; Cui et al., 2011). Injection inoculum was prepared to 100 colony forming units (cfu)/nl for all Mm experiments, which was injected into the circulation at 30 h post fertilisation (hpf) via the caudal vein.

Fungal infection experiments were performed using the *Candida albicans* strain TT21*-mCherry* (Seman et al., 2018). Overnight liquid cultures were grown from fungal plates, then prepared for injection as previously described (Seman et al., 2018). Cultures were counted using a haemocytometer and prepared in 10% PVP40 for 200 cfu/nl injection dose, which was injected into the circulation at 30 hpf via the caudal vein.

Fungal infection experiments were also performed using the *Cryptococcus neoformans* strain Kn99*-mCherry* (Gibson et al., 2018 preprint). Cryptococcal culture was performed as previously described (Bojarczuk et al., 2016) and, after counting on a haemocytometer, Kn99 was prepared in 10% PVP40 for 200 cfu/nl injection dose, which was injected into the circulation at 1-2 dpf.

### Microinjection of zebrafish larvae

Prior to injection, zebrafish were anaesthetised in 0.168 mg/ml tricaine in E3 medium and transferred onto 1% agarose in E3+Methylene Blue plates, removing excess medium. All pathogens were injected into the circulation to create systemic infection, using a microinjection rig (World Precision Instruments) attached to a dissecting microscope. A 10 mm graticule was used to measure 1 nl droplets for consistency, and droplets were tested every 5-10 fish and recalibrated if necessary. A final injection volume of 1 nl was injected to produce doses calculated for each pathogen. After injection, zebrafish were transferred to fresh E3 medium for recovery and maintained at 28°C.

### Whole-mount *in situ* hybridisation

RNA probes for zebrafish arginase type II (*arg2*, ENSDARG00000039269; plasmid obtained from Source Bioscience) were designed and synthesised after cloning into the pCR Blunt II-TOPO vector, according to the manufacturer's instructions (Thermo Fisher Scientific). Plasmids were linearised and probes synthesised according to the DIG RNA Labelling Kit (SP6/T7) (Roche). Zebrafish larvae were fixed in 4% paraformaldehyde solution (Thermo Fisher Scientific) overnight at 4°C. Whole-mount *in situ* hybridisation was performed as previously described (Thisse and Thisse, 2008).

### Confocal microscopy

Control, tailfin-transected and infected larvae were imaged using a Leica DMi8 SPE-TCS microscope using a HCX PL APO 40×/1,10 water immersion lens. For confocal microscopy, larvae were anaesthetised in 0.168 mg/ml tricaine and mounted in 1% low-melting agarose (Sigma-Aldrich) containing 0.168 mg/ml tricaine in 15 μ-Slide 4 well glass-bottomed slides (ibidi). Numerical data were determined using 40× confocal images.

### Stereo microscopy

Zebrafish larvae were anaesthetised in 0.168 mg/ml tricaine and transferred to a 50 mm glass-bottomed FluoroDish (ibidi). Zebrafish were imaged using a Leica DMi8 SPE-TCS microscope fitted with a Hamamatsu ORCA Flash 4.0 camera attachment using a HC FL PLAN 2.5×/0.07 and HC PLAN APO 20×/0.70 dry lens. Both transgenic zebrafish and whole-mount *in situ* staining was imaged using a Leica MZ10F stereo microscope fitted with a GXCAM-U3 series 5MP camera (GT Vision).

### Light-sheet microscopy

Larvae (2 and 3 dpf) were imaged using a Zeiss Z1 light-sheet microscope with Plan-Apochromat 20×/1.0 Corr nd=1.38 objective, dual-side illumination with online fusion and activated Pivot Scan at 28°C chamber incubation. Zebrafish were anaesthetised in 0.168 mg/ml tricaine and mounted vertically in 1% low-melting agarose in a glass capillary. Images were obtained using 16 bit image depth, 1400×1400 pixel field of view and GFP visualised with a 488 nm laser at 16% power, 49.94 ms exposure and user-defined *z*-stack depth (400-600 slices, 0.641 μm slices).

### Statistical analysis

Embryos/larvae were randomly assigned to experimental groups and experimenters were blinded to groups where possible. Sample size (*n* of larvae) was determined by the number of healthy embryos that were laid in the batch and number of groups within the experiment. As the *arg2:GFP* line was a new transgenic line, it was not possible to use power calculations pre-experimentation.

Microscopy data were analysed using Leica Application Suite X (LAS X; Leica Microsystems) and Image J software. All numerical data were analysed (Prism 9.0, GraphPad Software) using unpaired two-tailed *t*-tests for comparisons between two groups and one-way ANOVA (with Bonferroni post-test adjustment) for other data. *P*-values shown are: *P<0.05, **P<0.01 and ***P<0.001.

## Supplementary Material

10.1242/dmm.049966_sup1 Supplementary information
