# Supplementary material for: An arginase 2 promoter transgenic line illuminates immune cell polarisation in zebrafish
Source: Dis Model Mech. 2023 May 3;16(6):dmm049966. doi: 10.1242/dmm.049966 (PMC10163355; doi:10.1242/dmm.049966)
Supplement: Supplementary information [file dmm-16-049966-s1.pdf]

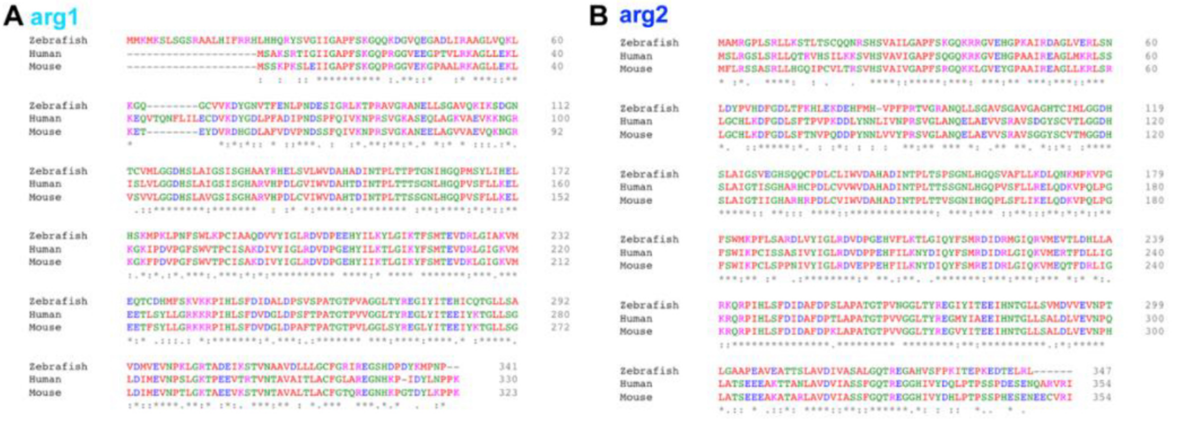

C

	Human/zebrafish cDNA Homology (% identity)	Human/zebrafish protein homology (% identify)	Human/zebrafish protein homology (% coverage)	Human/zebrafish peptide length (aa)
Arginase 1 (*arg1*, NM_001045197)	63/60	62/58	98/93	322/341
Arginase 2 (*arg2*, NM_199611)	60/61	60/61	97/99	354/347

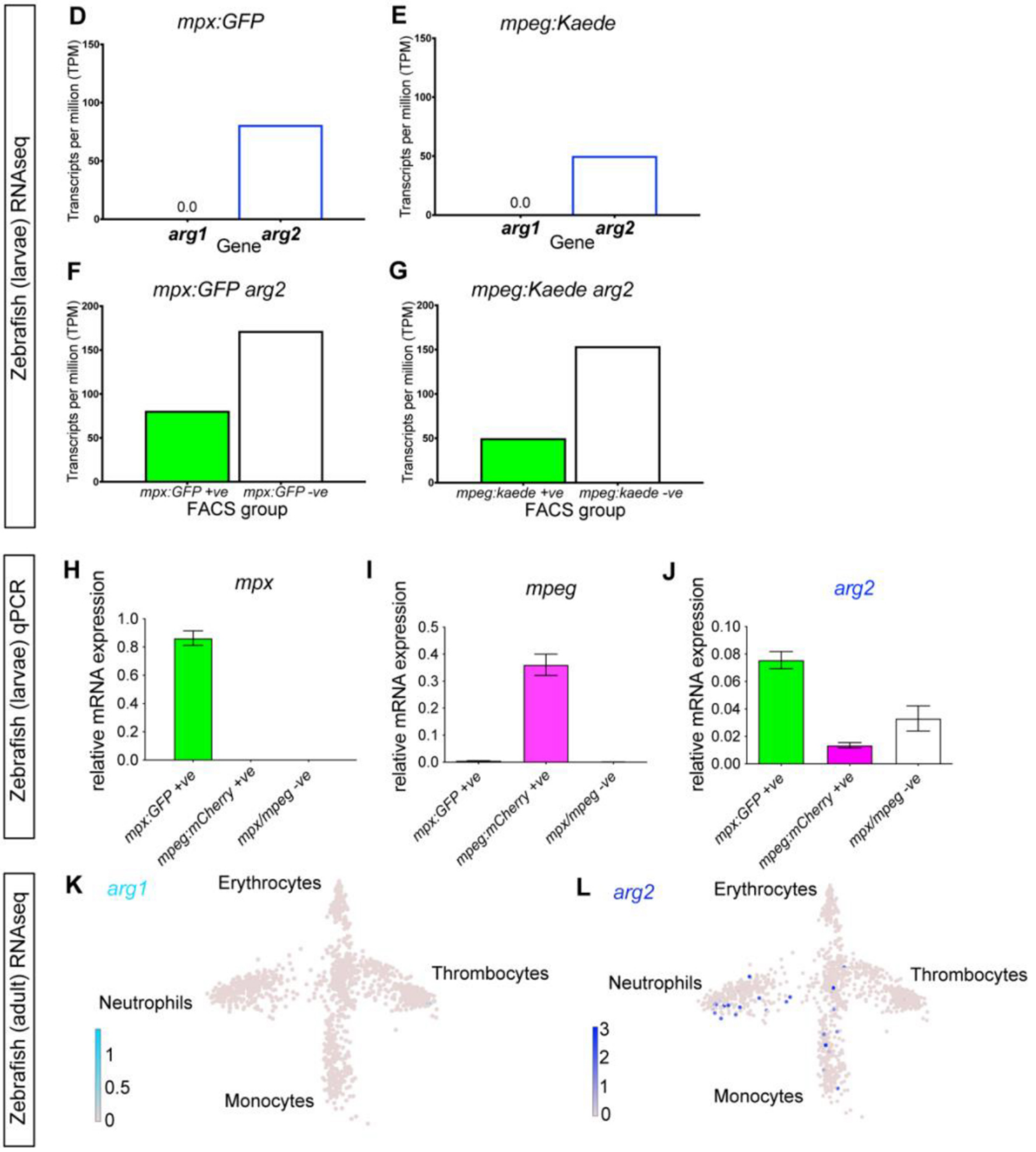

Disease Models & Mechanisms • Supplementary information

Fig. S1. Zebrafish leukocytes express more *arg2* than *arg1* in both larvae and adults

(A) Amino acid alignment of zebrafish, mouse and human Arginase 1.

(B) Amino acid alignment of zebrafish, mouse and human Arginase 2.

(C) Homology details of zebrafish and human Arginase 1 and Arginase 2.

(D-G) Zebrafish neutrophils sorted via FACS of ~30 pooled 5dpf *Tg(mpx:GFP)i114* larvae and RNAseq performed on *mpx:GFP*+ (neutrophils, D and F) or *mpx:GFP*- (rest of fish) cells (E). Zebrafish macrophages sorted via FACS of ~30 pooled 5 days post fertilisation (dpf) *Tg(mpeg1:Gal4-VP16)gl24/(UAS-E1b:Kaede)s1999t* larvae and RNAseq performed on *mpeg:Kaede*+ (macrophages, D and F) or *mpeg:Kaede*- (rest of fish) cells (E). Datasets obtained from Rougeout *et al*., 2019. Data shown is an average value from 3 replicates.

(H) Relative expression of *mpx* gene compared to house-keeping gene *ef1a*, obtained by RT-qPCR on FACS isolated neutrophils (*mpx:GFP* positive), macrophages (*mpeg:mCherry* positive) and the rest of the tissues (*mpx/mpeg* negative) from 3dpf larvae.

(I) Relative expression of *mpeg* gene compared to house-keeping gene *ef1a*, obtained by RT-qPCR on FACS isolated neutrophils (*mpx:GFP* positive), macrophages (*mpeg:mCherry* positive) and the rest of the tissues (*mpx/mpeg* negative) from 3dpf larvae.

(J) Relative expression of *arg2* gene compared to house-keeping gene *ef1a*, obtained by RT-qPCR on FACS isolated neutrophils (*mpx:GFP* positive), macrophages (*mpeg:mCherry* positive) and the rest of the tissues (*mpx/mpeg* negative) from 3dpf larvae.

(K-L) Gene expression of adult zebrafish leukocytes was determined using the zebrafish blood atlas (Athanasiadis *et al*., 2017, (Athanasiadis et al., 2017)). Each point represents separate scRNAseq replicates performed across multiple zebrafish wildtype and transgenic strains. Each arm of schematic indicates separate blood cell population (labelled). Deeper colour indicates higher expression (log10 scale bars described for each gene).

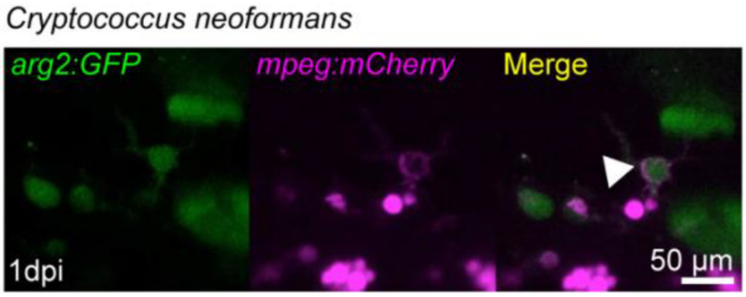

Fig. S2. Macrophage *arg2:GFP* expression at 1dpi with *Cryptococcus neoformans*
Fluorescence confocal micrographs of *arg2:GFP* crossed to *mpeg:mCherry* after *Cryptococcus neoformans* infection at 1dpi with *arg2:GFP* positive macrophage indicated by arrowhead.

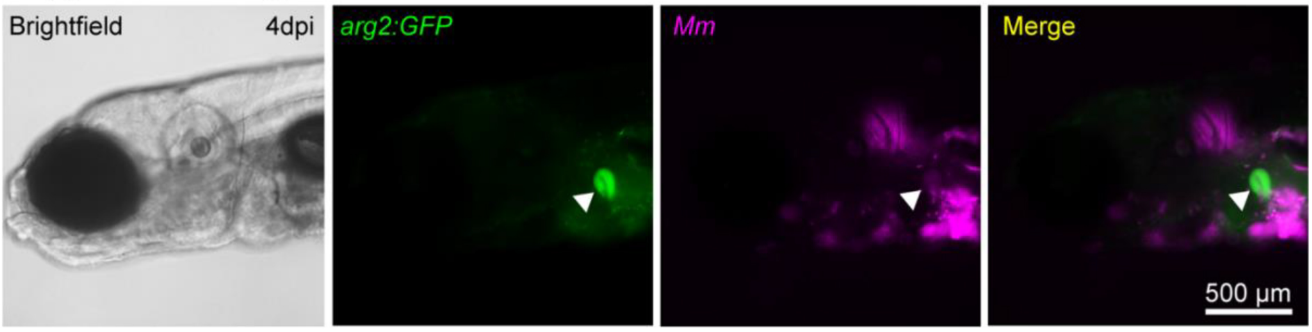

Fig. S3. Mm infection upregulates *arg2:GFP* in the liver
Brightfield and fluorescence micrographs of *TgBAC(arg2:GFP)sh571* larvae at 4dpi after Mm infection showing *arg2:GFP* liver expression with heavy levels of infection.
